# Context Breeds False Memories for Indeterminate Sentences

**DOI:** 10.3389/fpsyg.2021.616065

**Published:** 2021-03-12

**Authors:** Levi Riven, Roberto G. de Almeida

**Affiliations:** Department of Psychology, Concordia University, Montreal, QC, Canada

**Keywords:** indeterminate sentences, compositionality, false memory, pragmatics, inferences, semantic coercion, propositional representation, sentence comprehension

## Abstract

What are the roles of semantic and pragmatic processes in the interpretation of sentences in context? And how do we attain such interpretations when sentences are deemed indeterminate? Consider a sentence such as “*Lisa began the book*” which does not overtly express the activity that Lisa began doing with the book. Although it is believed that individuals compute a specified event to enrich the sentential representation – yielding, e.g., “*began [reading] the book*” – there is no evidence that a default event meaning is attained. Moreover, if indeterminate sentences are enriched, it is not clear where the information required to generate enriched interpretations come from. Experiment 1 showed that, in isolation, there is no default interpretation for indeterminate sentences. The experiment also showed that biasing contexts constrain event interpretations and improve plausibility judgments, suggesting that event representations for indeterminate sentences are generated by context. In Experiment 2, participants heard biasing discourse contexts and later falsely recognized foil sentences containing the biased events (“*Lisa began reading the book*”) at the same proportion and with the same confidence as the original indeterminate sentence (“*Lisa began the book*”). We suggest that indeterminate sentences trigger event-enriching inferences but only in sufficiently constraining contexts. We also suggest that indeterminate sentences create two memory traces, one for the proposition consistent with the denotational, compositional meaning, and another for the proposition that is enriched pragmatically over time.

## Introduction

We are rarely faced with the task of understanding sentences in isolation. Most often, linguistic expressions are understood and produced in rich utterance contexts, allowing us to interpret rather easily a variety of incomplete or anomalous expressions, such as disfluencies (*Well…uh…she…left!*) and metaphors (*He’s a pig!*) – which are literally false but invite us to seek alternative interpretations. An arguably more subtle case is that of sentences deemed indeterminate or underspecified. For instance, if you were told upon breaking into a sneezing fit, *Do not worry, you are not going to die*, you would not likely interpret this sentence as declaring your immortality, nor would you think it is false. Rather, you would take the sentence to convey that you are not going to die *as a consequence of* your sneezing. Such indeterminacies are ubiquitous in natural language and are generally taken to be resolvable by simply filling-in the “blanks” – or what Perry (1986) called “unarticulated constituents” – with information supplied by context or by some default semantic operation.

Here, we report on two experiments investigating the interpretations that participants assign to indeterminate sentences in isolation and in context. The phenomenon we investigated, more specifically, involves sentences such as (1):

1. Lisa began the book.

While it is clear that Lisa began doing something with a book, the sentence is indeterminate with regards to what exactly Lisa began doing. This kind of construction has been the object of investigation in theoretical linguistics (Pustejovsky, 1995, 2011; de Almeida and Dwivedi, 2008; de Almeida and Riven, 2012; Asher, 2015; see also de Swart, 2011, for review), psycholinguistics (e.g., McElree et al., 2001; Traxler et al., 2002; de Almeida, 2004), and neuroimaging (Pylkkänen and McElree, 2007; Husband et al., 2011; de Almeida et al., 2016). What motivates general interest in indeterminate sentences is that, although they are grammatical and semantically felicitous – for a truth value judgment *can* be made – they appear to convey more information than what is explicitly said. Specifically, a sentence such as (1) in isolation is compatible with a wide range of events – such as *reading*, *writing*, *burning*, or even *eating* – each of which might serve as a suitable interpretation. However, it is not clear what exactly might be attained, nor which linguistic and cognitive systems are deployed in attempting to resolve indeterminacy.

Thus far, there have been several proposals on how these sentences are interpreted in isolation and in context. These proposals vary along several dimensions, including the degree to which the sentence is enriched, the role of context in the enrichment process, the formal mechanisms for this enrichment, and the source of information employed in enriching indeterminate sentences (e.g., Pustejovsky, 1995, 2011; de Almeida and Dwivedi, 2008; Pylkkänen, 2008; Asher, 2015). Some proposals appeal to *type-shifting* rules which work to *shift* the semantic types of constituents to allow for semantic composition – thus making the verb and noun-phrase complement “fit” together. Other proposals go beyond, appealing to a form of lexical-semantic *interpolation* – viz., by augmenting the proposition that a sentence such as (1) conveys, yielding a particular event in the resulting proposition, similar to what would be conveyed by (2).^1^

2. Lisa began *reading* the book.

We briefly discuss some of these proposals below, but suffice it to say now that, for versions of the *interpolation* view (e.g., Pustejovsky, 1995, 2011; Lapata and Lascarides, 2003; Traxler et al., 2005) the meaning of (1) might be quasi-synonymous with (2). To a large extent, this assumption follows from the earliest studies of indeterminacy which proposed that sentences such as (1) appear almost as frequently in corpora as fully-determinate variants such as (2) (e.g., Briscoe et al., 1990). Moreover, an early empirical study found that participants rate indeterminate sentences as being just as sensible as their fully determined controls (McElree et al., 2001). Such findings suggest that indeterminate sentences trigger a systematic enrichment process that is believed to resolve indeterminacy, thus allowing for frequent and felicitous use in natural speech contexts.

This enrichment process has since been the object of considerable experimental investigation involving numerous techniques such as self-paced reading (McElree et al., 2001; de Almeida, 2004), eye-tracking (Traxler et al., 2002, 2005; Pickering et al., 2005; McElree et al., 2006a; Frisson and McElree, 2008; Katsika et al., 2012; Zarcone et al., 2014; Antal and de Almeida, 2020), probe recognition (Zarcone et al., 2014), sensibility judgments (McElree et al., 2006b), event-related potentials (ERPs; Baggio et al., 2010; Kuperberg et al., 2010), fMRI (Husband et al., 2011; de Almeida et al., 2016), magnetoencephalography (MEG; Pylkkänen and McElree, 2007), and aphasia (Piñango and Zurif, 2001). The majority of these studies have shown that indeterminate sentences engender processing delays or different activation patterns relative to various types of control sentences. The greater processing costs of indeterminate sentences are thought to correspond to the mental operations associated with *semantic* enrichment of sentences such as (1), leading to the creation of a proposition that corresponds to the content of (2).

However, several observations from recent reports cast doubt on the assumption that indeterminate sentences are fully enriched during online processing. At least four studies have reported plausibility norms for which indeterminate sentences were rated statistically lower than controls (Pylkkänen and McElree, 2007; Frisson and McElree, 2008; Katsika et al., 2012; de Almeida et al., 2016). In addition, one study that measured acceptability ratings incrementally, as sentences were being presented, showed that participants rate indeterminate sentences as unacceptable, suggesting that they might not achieve a fully specified event interpretation (McElree et al., 2006b).^2^

The vast majority of psycholinguistic experiments have emphasized processing times, including response times (RTs) and eye-movement behaviors, over other measures of interpretation, and have thus left open the question as to what sort of interpretation indeterminate sentences actually get. While the processing delays associated with such sentences are thought to correspond with some form of enrichment-related process, it may be the case that individuals’ interpretations of indeterminate sentences are left indeterminate and in fact look more like (1) than (2). In other words, differences in RTs do not *per se* point to enrichment (de Almeida and Dwivedi, 2008) and, if they do, it is far from clear how sentences might be enriched.

Studies involving more direct measures of brain activity such as blood flow (fMRI) and electrical signals (ERP and MEG) have also consistently showed differences between indeterminate sentences and their controls, and have contributed to an understanding of functional and neuroanatomical resources involved in interpreting these sentences. But these techniques are also limited with regards to what information is attained when sentences are understood. One advantage of neuronal recording techniques (MEG and ERPs) over RTs and fMRI is that neuronal recordings yield information about the time-course of events associated with processing of indeterminate sentences. The study by Kuperberg et al. (2010), for instance, found a small but significant N400 effect in the contrast between indeterminate and control sentences in two grand-averaged sites. But similar to RT and MEG studies, these commonly found differences still call for an explanatory framework as to what sort of *content* comes into play. In the case of fMRI studies, results have also been far from conclusive. For instance, one study (Husband et al., 2011) supports a mandatory *type-shifting* effect, based on greater left-frontal and left-temporal activation for indeterminate sentences. Another study (de Almeida et al., 2016) calls for a pragmatic, inferential resolution, based on greater whole-brain activation, and in particular greater right-hemisphere and anterior cingulate cortex (ACC) activation, for indeterminate sentences, signaling a greater search for an interpretation. While the details of all their analyses are beyond the focus of the present paper, it is important to note that, as with other techniques, fMRI also does not allow for a clear understanding of what sort of content indeterminate sentences call for and when they are enriched.

We thus sought to investigate, in the present study, the nature of the *propositional content* elicited by indeterminate sentences in context and over time. Contrary to the studies briefly mentioned above, we were primarily focused on understanding what is retained in memory, both immediately and after a delay. We were motivated, in particular, by the classic effect of “gist” first obtained by Sachs (1967, 1974). In those studies, Sachs showed that participants quickly forget the *verbatim* form of a sentence and falsely recognize foils if these preserve the gist – i.e., propositional content – of the original stimulus sentence. Our goal was to go beyond this effect and determine if subjects would falsely recognize *Lisa began reading the book* (2) as being the originally presented *Lisa began the book* (1), within a biasing “book reading” context. While one would hardly dispute that discourse context exerts an influence on how one might ultimately interpret a sentence (see, e.g., O’Brien and Cook, 2015, for a review), our goal was to focus on the propositional content that is obtained as an indeterminate sentence and is heard and what becomes of this proposition in memory over time. Thus, beyond response times and neuronal recordings – which have provided us important insights into the processing of indeterminate sentences in isolation (but see de Almeida, 2004 and Traxler et al., 2005) – our goal was to focus on the nature of the proposition encoded in memory, in context, and over time.

### (How) Are Indeterminate Sentences Enriched?

In order to further elaborate on the questions that motivate our study, it is important to examine how we might interpret indeterminate sentences in context. It seems clear that, if a sentence such as (1) is uttered in a discourse context related to reading, it may convey information that is compatible with what (2) says. Clearly, however, the two sentences – (1) and (2) – are not synonymous, even if the intention of the speaker of (1) is to communicate that Lisa began *reading* a book. The truth conditions of the two sentences also differ radically, because (2) but not (1) is true only if Lisa began *reading* a book, whereas (1) is true no matter what Lisa began doing with the book. Thus, while (1) and (2), on the surface, convey two different propositions, interpolation proposals for enrichment would assume that the proposition conveyed by (1) is actually modified to convey something different from what its meaning communicates at face value – something akin to (2). The studies mentioned above, involving several reading-time measures (self-paced reading and eye-tracking) as well as those involving ERP and MEG, have in fact indicated that the process of enrichment occurs rapidly, at or right after the processing of the complement noun (*book*). This suggests that the proposition conveyed by (1) is possibly fully formed as something like (2) by *default* (Pustejovsky, 1995, 2011; Lapata et al., 2003). While this is a viable outcome of how enrichment might work, there is no direct experimental evidence for this process other than differences in RTs and activation patterns, as discussed above, which are compatible with several theoretical explanations.

One such explanation attributes the greater processing cost of indeterminate sentences to the process of *type-shifting* the complement noun from an *entity* complement (*book*) to an *event* performed with the referent of the noun complement. The standard version of this theory (e.g., Partee, 1986) proposes a basic set of semantic types for noun phrases (NPs; e.g., *quantificational*, *entity/referential*, and *predicative*) with these NPs changing (viz., *shifting* – such as *lowering* or *lifting*) their types according to the requirements of the verb in order to semantically compose. An aspectual verb such as *begin*, by hypothesis, requires an *event* complement. Given that the NP *the book* may have a default *entity* type, it shifts to an *event* to compose with the verb. A type-shifting rule is not, in principle, a form of content enrichment – that is, it does not provide content to the resulting proposition other than changing the reading (thus, the computation) of the *entity* nominal into an *event* to allow for semantic composition.

Perhaps the most influential view in this camp is that of *generative lexicon* theory (GL; Pustejovsky, 1995, 2011; see also Pustejovsky and Batiukova, 2019, for a recent review). GL proposes a greater variety of semantic *types* together with information that lexical representations (“qualia structure”) encode about the meanings of nouns. The mechanism for how indeterminate sentences are enriched, more specifically, involves at least two component processes: (1) the retrieval of an event such as *reading*, supposedly stored within the conceptual representation of the noun *book* (the “qualia structure”); and (2) interpolation of the retrieved event into the indeterminate verb phrase (VP) *began [reading] the book*. Although this view has undergone several changes over the years, the basic mechanisms stand: in its more current version, GL proposes at first a form of type-shifting that relies on the subtypes carried by nominals. These subtypes (e.g., *physical* and *information*) are part of the qualia structure of the lexical entry and are associated with particular roles, such as *telic* (the purpose; e.g., *read* for *book*). In such cases, one of the operations involves selecting one of the subtypes for the noun complement and recover the role from its qualia structure. This qualia information is then used to enrich the representation of the sentence, adding semantic material to its logical form, that is, providing “a potential (default) interpretation for the predicate associated with the event” (Pustejovsky, 2011, p. 1422). In the GL framework, then, the analysis of a sentence such as (1) involves (i) the detection of a mismatch between the verbs’ restrictions (viz., requiring an *event* complement) and the semantic type of the complement, followed by (ii) the insertion or *interpolation* of a plausible *event* to yield an enriched semantic composition similar to what sentence (2) conveys.

Although initial evidence of processing delays was taken to support this form of coercion with interpolation (McElree et al., 2001; Traxler et al., 2005; but see de Almeida, 2004), later psycholinguistic experiments suggested that coercion does not necessarily entail the retrieval of a specified event. Using a speed-accuracy trade-off paradigm, McElree et al. (2006b) proposed that participants do not build fully specified event interpretations. In a paradigm that involved incremental acceptability ratings of unfolding sentences, they showed that, while participants were slower to respond to indeterminate sentences like (3a) compared to controls like (3b), they maintained low acceptability ratings for several seconds after the sentence was presented, suggesting that the extra effort associated with processing these sentences did not deliver a fully enriched interpretation.

3.
  - The carpenter began the table.
  - The carpenter built the table.

These findings support type coercion without interpolation, that is, without necessarily activating a conceptual representation of the activity *per se* (e.g., *reading* and *building*). This implies that enriched event conceptualizations – i.e., those denoting specific activities – are not achieved autonomously from the lexical entry of the noun. However, they do not preclude the possibility that readers may access event interpretations *pragmatically* (viz., by deploying inferential processes) when additional constraints are presented to ease interpretation, such as a supportive discourse context.

In an experiment exploring the role of pragmatic constraints on processing times, de Almeida (2004) presented participants with short discourse contexts such as (4a), which were designed to activate knowledge about the type of events that are likely to unfold. These passages were followed by either an indeterminate sentence such as (4b), a preferred control such as (4c), or a non-preferred control such as (4d).^3^

4.
  - The secretary would always be sure to work ahead of schedule. She was asked to work on a memo.
  - The secretary *began* the memo long before it was due.
  - The secretary *typed* the memo long before it was due.
  - The secretary *read* the memo long before it was due.

Previous research had shown that when these indeterminate sentences are presented without context, they are costly relative to both preferred and non-preferred controls (McElree et al., 2001). However, de Almeida found that when context was provided, RTs to indeterminate sentences were slower compared to preferred controls only, and were equivalent to non-preferred sentences. The finding suggests that processing delays might reflect pragmatic-inferential means of enrichment (see Fodor and Lepore, 2002; de Almeida and Lepore, 2018). That is, indeterminate sentences may be more costly in isolation than in context because enrichment requires readers to draw local inferences about possible event interpretations. And readers may fail to retrieve event meanings when no contextual support is provided. Context, however, serves to further constrain interpretations – yielding indeterminate representations on a par with non-preferred interpretations.

This pragmatic proposal, more explicitly, takes the view that an early linguistic analysis of the sentence yields an *unenriched* proposition, one that is left indeterminate and is compatible with the sentence input. The proposal takes an indeterminate sentence such as (1), above, to be grammatical and felicitous, not one that is semantically defective. It assumes that any further enrichment of the initial proposition comes as a function of inferences triggered by the proposition, taking into account all possible sources of information but most importantly the context of the utterance. Crucially, the pragmatic hypothesis makes two proposals that set it apart from the interpolation view: first, the initial proposition is attained as a translation of the input, with no enrichment *by necessity*; and, second, any enrichment is a natural consequence of causal inferential processes, not by appealing to internal analyses of word meanings or what has been called semantic decomposition (see Fodor and Lepore, 2002; de Almeida and Dwivedi, 2008; de Almeida and Riven, 2012; de Almeida and Lepore, 2018; de Almeida and Antal, 2020). This hypothesis, therefore, places the burden of enrichment – if anywhere – on inferential pragmatics, not on local default semantic processes.

Note that there are different views within the “pragmatic” camp on how the composition of (1) is achieved. For some (Fodor and Lepore, 2012), (1) means that Lisa began doing something with the book. This view relies on the notion that lexical concepts are *atomic* (i.e., nondecompositional) but also carry information about how they combine with other lexical concepts when they compose into propositions. For others (de Almeida and Dwivedi, 2008; de Almeida and Riven, 2012; de Almeida and Lepore, 2018), the *something* that Lisa began doing with the book is syntactically determined, that is, having a more complex VP – a syntactic representation that introduces a phonologically and morphologically empty *verb* node, one that is licensed by the aspectual verb. This node, although not filled semantically, operates as a processing trigger to generate pragmatic inferences about possible events [see de Almeida and Dwivedi, 2008 and de Almeida and Riven, 2012, for details on the syntactic analysis of sentences such as (1)].

It is also important to note that these views are in general agreement with the type-shifting (without interpolation) idea that a sentence such as *Lisa began the book* yields a different computation than a fully determined sentence such as *Lisa read the book*. Both camps assume that the former requires an analysis of the VP – and in particular the relation between the main verb and the complement NP – that relies on either the specification of a syntactic node, more lexical structure, or a semantic type-shifting operation on the complement NP. They thus agree that what Lisa began was an event with the book. In addition, both pragmatic and type-shifting views agree that however the sentence is analyzed, there is no *interpolation* of specified events by default.

A different but compatible proposal, “words-as-cues” (Zarcone et al., 2014) also assumes that inferences about events are generated online, not *via* mandatory semantic operations, with discourse or co-textual lexical units providing clues on what the event is most likely to be about. This proposal – billed as in-between the type-shifting and pragmatic hypotheses – takes the resolution of indeterminate sentences to be a function of the activation of multiple constraints, giving context the role of what the pragmatic theory assumes to be, roughly, that of inferences. It is rather difficult to fully contrast the “words-as-clues” with the pragmatic proposal on grounds that they are committed to different cognitive architectures. That is, while the pragmatic proposal makes a distinction between linguistic (viz., syntactic/semantic) level and pragmatic inferences, the word-as-cues hypothesis is committed to a connectionist/interactive-activation framework, which makes no clear distinctions between lexical units, what they mean (concepts), the propositions they partake (namely, how they compose meaning), and the inferences they trigger. Nonetheless, the view of Zarcone et al. is that information obtained to interpret the complement noun is general, encyclopaedic information, not linguistic *per se*, a view that is compatible with the pragmatic proposal for enrichment.

Those who support the interpolation hypothesis agree in principle that pragmatic inferences contribute to the enrichment process (e.g., Traxler et al., 2005) and, in fact, assume that co-text and context play a role in suggesting interpretations for the indeterminate VP. For instance, in an offline sentence completion task (Lapata et al., 2003), participants more frequently produced *writing* for a sentence fragment such as *The author began ___ the book*, containing an *agentive* subject – i.e., one that specifies the creation of the entity denoted by the noun. But when the agent was *The student*, participants more frequently produced *reading*, thus more in agreement with a *telic* subject – i.e., one that specifies one possible purpose of the entity denoted by the noun. In a neutral context, when the agent was a proper name, however, *reading* was produced more frequently than *writing*, suggesting that the *telic* information (or *role*) is the default event meaning associated with the entity. Lapata et al. suggested that the verb also provides information that might determine the interpretation of the whole verb phrase, that is, in combination with its complement noun, with cases in which a telic interpretation might be preferred (*endure the speech*), others in which an agentive interpretation might be preferred (*regret the speech*) and others in which there is no default (*enjoy the speech*). What these cases seem to show, in fact, is that the hypothesis of default interpretation is highly specific to contexts or situations beyond the information that the agent and complement provide.

Besides, the very idea that the subject NP suggests that what the author began doing is *writing* the book, just begs the question as to how this operation takes place in consonant with the interpolation process. Traxler et al. (2005) suggest that two key processes are triggered by the alleged mismatch between the selectional restrictions of the verb (*begin*) and the complement noun (*book*). According to them,

“Comprehenders use salient properties associated with the complement noun and other relevant discourse elements (including but not necessarily limited to the agent phrase) to infer a plausible action that could be performed on the noun (Traxler et al., 2005, p. 4).”

And, further,

“Comprehenders incorporate the event sense into their semantic interpretation of the VP by reconfiguring the semantic representation of the complement, converting [*_β_* began (*_α_* the book)] into [*_β_* began (*_α_* reading the book)]. (Conceivably, this could also require reconfiguration of an associated syntactic representation)” (p. 4).

If we understand this proposal well, these processes call for both, (a) inferences on potential actions performed by the subject over the object, but also (b) an actual semantic interpolation by a chosen activity. Crucial to the present discussion is the role attributed to pragmatics. As with the proposal of Zarcone et al. (2014), where there appears to be no distinct levels of representation for activated units, the proposal of Traxler et al. puts all processes bearing on content (lexical-semantic and pragmatic) in the service of an enriched semantic composition – and with operations that appear to be over *sentences* (viz., syntactic reconfiguration), not propositions.

While the hypothesis that contextual information beyond sentence constituents may play a key role in the interpretation of indeterminate sentences, thus far few studies since de Almeida (2004) have investigated contextual influence on interpretation directly, and evidence for pragmatic enrichment remains mixed. Traxler et al. (2005), for instance, also manipulated discourse context and showed that including the to-be-inferred event in the immediate discourse did not consistently reduce processing times for indeterminate sentences. For instance, in their Experiment 1, participants were shown passages that either suggested an event such as in (5a) or were neutral, as in (5b), with both followed by an indeterminate sentence (5c) or a control (5d).

5.
  - The contractor had been building in the suburbs.
  - The contractor had been looking for new jobs.
  - That spring, he began a condominium …
  - The spring, he built a condominium …

Supporting the interpolation view, Traxler et al. report several analyses in which reading times for the indeterminate sentence are longer than the control in contexts such as (5a), suggesting that coercion is mandatory, despite contextual information providing an event interpretation (*building*) for the indeterminate sentence. But their results are difficult to interpret as unequivocal support for interpolation, for several reasons. First, their effects were not consistent across experiments manipulating similar, but slightly modified materials. Second, most effects appeared in the verb region rather than in the crucial post-verbal regions. And third, many of the effects were either statistical tendencies or marginal, even when measures of reading time were relatively late, such as in regressions and re-reading times (see, e.g., Traxler, et al., 2005, Experiment 3). Also important is an effect in their Experiment 2 showing greater total fixation times for indeterminate sentences [as in (5c)] at the noun complement region when these sentences followed neutral contexts [as in (5b)] than when indeterminate sentences followed event contexts such as (5a). This is what the pragmatic theory would predict: when an event is suggested by the context, it provides the indeterminate sentence with a possible interpretation; in the absence of such a suggestive context, indeterminate sentences may trigger pragmatic inferences, thus yielding greater costs (see de Almeida and Dwivedi, 2008).

Most RT experiments suggest that indeterminate sentences are difficult to process, but that this difficulty is not necessarily associated with an event retrieval process, be it *via* semantic interpolation or pragmatic inferences. Although the prevailing view is that sentence-enriching inferences occur autonomously and in a cost-free manner (Traxler et al., 2005; Frisson and McElree, 2008), there is no evidence that participants’ interpretations actually include such interpolated representations. In fact, there seems to be growing evidence to the contrary in the form of plausibility, acceptability, and cloze norms. As we noted above, a growing number of studies reporting offline norming tasks corroborate McElree et al. (2006b) result of lower acceptability ratings. Although plausibility norms for these indeterminate sentences are consistently higher than anomalous sentences, they are often statistically lower than control sentences (Pylkkänen and McElree, 2007; Frisson and McElree, 2008; Katsika et al., 2012; de Almeida et al., 2016). And, occasionally, comprehension checks suggest that indeterminate sentences are interpreted less accurately (Husband et al., 2011). Even cloze tasks yield differences (Kuperberg et al., 2010) – with, for instance, subjects producing the complement noun (“*article*”) significantly more for frames such as *The journalist wrote the…* than for *The journalist began the…*.

All together, these data are difficult to reconcile with the view that an event interpretation is automatic, deploying some default interpolation operation. Poor comprehension metrics suggest that there is uncertainty about the events that indeterminate sentences are intended to convey and that they are likely not enriched by a mechanism that involves augmenting the proposition. If event information comes into play at all, it is presumably contingent on the availability of inferential cues within the broader utterance context (de Almeida and Lepore, 2018).

### The Present Study

In order to disentangle these issues, we investigated the representation attained in memory for indeterminate sentences and the role that the contextual information plays in possibly suggesting interpretations for these sentences. Experiment 1 addressed the question of whether lower cloze probabilities and acceptability ratings of indeterminate sentences, as obtained in several empirical studies (e.g., McElree et al., 2006b; Kuperberg et al., 2010; Katsika et al., 2012; de Almeida et al., 2016), can be attributed to the absence of a supportive discourse context. Moreover, we were interested in obtaining a “default” meaning as in Lapata et al.’s (2003) neutral condition. In our Experiment 2, then, we further investigated whether contextualized indeterminate sentences trigger event interpretations, using a long-term memory (LTM) recognition paradigm that relies on recovering the propositional content of sentences (Sachs, 1967, 1974). Crucially, Experiment 2 aimed at tracing the “gist” or proposition obtained at the moment the original indeterminate sentence was presented and later, when it had been consolidated in LTM. We reasoned that probing at different points during the presentation of the discourse, as first done by the classic Sachs experiments, could give us information on how context might influence interpretation and thus potentially create false memories, beyond the immediate encoding of the indeterminate sentence.

It is important to stress that it is not under question in the present investigation whether or not context influences how we ultimately attain a particular meaning for a sentence. There are numerous sources of evidence for the influence of context on the interpretation of words and sentences. It is clear – to mention a classic example – that in cases of lexical ambiguity (Swinney, 1979; Tabossi and Zardon, 1993) sentential and even wider context help determine which meaning is attained, even when initially all possibilities are entertained. And it has been amply demonstrated that information in a text is constantly generating inferences during reading – both local and global – that continually aid in the comprehension of sentences and of these in relation to global discourse (McKoon and Ratcliff, 1988, 1992; see also Kintsch, 1998 and O’Brien and Cook, 2015, for a recent review). Moreover, it has also been demonstrated that attention to sentences and discourse is not always accurate, leading to many interpretation errors that are taken to be at odds with the idea that we are constantly composing meaning that is faithful with the input stimuli (Sachs, 1967; Christianson et al., 2001; Sanford, 2002). But what is not clear is *how* context exerts its effects, that is, how the *proposition* that a sentence conveys is affected by context – whether enriched or impoverished – nor what is ultimately attained when an indeterminate sentence is processed in isolation and in a biasing context. And while we expect discourse to fully propose a resolution for a sentence deemed indeterminate, it is also not clear what happens with the memory trace of the original proposition – whether it is discarded and replaced by one that is supported by context.

## Experiment 1

In this experiment, we examined the role of context in generating event interpretations and acceptability of indeterminate sentences. Typically, online processing studies of indeterminate sentences aim to match experimental and control sentences on measures of plausibility and/or comprehension to validate materials. In several studies, indeterminate sentences have been rated or interpreted on par with controls (McElree et al., 2001; Traxler et al., 2002; Pickering et al., 2005), but they are frequently considered less felicitous (Pylkkänen and McElree, 2007; Frisson and McElree, 2008; Husband et al., 2011; Katsika et al., 2012; de Almeida et al., 2016). Could their relatively low acceptability result from uncertainty about the events that indeterminate VPs refer to? And would participants provide a “default” filler, as obtained by Lapata et al. (2003)? To address these questions, we presented participants with indeterminate sentences in isolation and within discourse contexts in order to (a) evaluate the convergence of event interpretations that participants generate in a fill-in-the-blank task, and (b) measure their acceptability ratings. We hypothesized that embedding sentences within discourse contexts would, first, increase the proportion of participants providing the same event verb in a fill-in-the-blank task; and, second, context would improve acceptability ratings of indeterminate sentences to the level of fully specified control sentences.

### Method

#### Participants

A total of 120 Concordia University undergraduate students divided into four groups participated in two between-subject tasks. Sixty students participated in the fill-in-the-blank task (42 in the no-context condition and 18 in the context condition) and 70 students participated in the ratings task (20 in the no-context condition and 50 in the context condition). All participants were native speakers of English and were compensated with course credit. They all gave informed written consent. The experiment was approved by the Concordia University Human Research Ethics Committee.

#### Materials and Procedure

##### Fill-in-the-Blank Task

A set of 19 indeterminate sentence frames such as *Lisa began ______ the book* were presented interspersed with 45 filler sentences lacking a verb such as *The cow _____ the field*. We presented participants with these frames on an Excel spreadsheet, leaving blank the column corresponding to the to-be-filled portion of the sentence. Participants were required to type a word that best completed the sentence, by filling the slot (see Supplementary Material S1 for full instructions given to participants). All experimental sentences had proper names as subjects to prevent agent-patient semantic associations that could confound the source of the information – i.e., as to whether events were generated from the sentential context or from the broader discourse context. This manipulation was, thus, equivalent to the neutral condition in Lapata et al. (2003). These sentences were presented in isolation (no-context condition) to 42 participants. Another group of 19 participants were presented with the same sentences when these were preceded by a four-sentence paragraph (see Preceding Context in Table 1).

**Table 1:** Sample materials employed in Experiments 1 and 2.

Condition/sentence type	Example
Preceding context	Lisa had been looking forward to the new Grisham novel ever since it came out. She had finally managed to set aside some time this weekend and made sure to make her home library nice and cozy. First thing Saturday morning, Lisa curled up on the sofa in her library with a blanket and a fresh cup of coffee. With everything in place, …
Indeterminate	Lisa began the book.
Biased foil/full-VP	Lisa began reading the book.
Non-biased foil	Lisa began writing the book.
Following neutral discourse	▲Suddenly, the doorbell rang. Lisa grunted, put down her coffee, and sluggishly made her way to the door. It was her neighbor John and he was out of peanut butter again. Looking through the cupboard, Lisa realized she was no better off. She told John that he was out of luck and suggested he try calling Mary, their mutual neighbor.▲

▲: Locations of visual probe presentation in Experiment 2.

The contexts were designed to generate an event schema, specifically by establishing the agent’s intention to perform a target activity – in this case *reading* – without mentioning the verb, neither before nor after the presentation of the target sentence. In both the no-context and context conditions, participants were instructed to provide a plausible verb to complete each sentence. See Supplementary Material S2, items 1–19, for the materials used in this task.

##### Rating Task

Five additional sentences were added to the set of materials described above (see Supplementary Material S2, items 20–24) and all 24 items were used for ratings. The ratings task also had two parts, one with sentences in isolation (no-context condition) and one with each sentence preceded by a context. In the no-context condition, 24 indeterminate sentences were presented interspersed with 39 filler sentences for a total of 63 sentences. All 20 participants in the no-context condition saw the same set of 63 sentence materials.

In the contextual condition, indeterminate sentences were presented as the concluding clause of passages. For this condition, 50 participants were divided into five orthogonal lists that included an equal number of indeterminate sentences and full-VP sentences. The full-VP sentences included the event interpretation that was constrained by the context (see Table 1). These contextualized sentences were interspersed with 33 filler paragraphs.

In both the no-context and contextual conditions, participants were given a printed booklet and were instructed to rate the plausibility of each sentence on a 5-point scale, with higher ratings indicating greater plausibility (see Supplementary Material S1, for full instructions).

### Results and Discussion

All context/no-context comparisons were analyzed by items using Welche’s two-sample *t*-test in the “stats” package in R (R Core Team, 2014). In the fill-in-the-blank task, the mean proportion (*p*) of participants that generated the dominant verb increased by 22% when indeterminate sentences were embedded in a biasing context, *t*(18) = 3.43, *p* < 0.001, *d* = 0.53 (see Table 2 for means and SDs). Thus, context constrained the range of event interpretations that participants assigned to indeterminate sentences. Context also improved plausibility ratings. Specifically, ratings were higher for indeterminate sentences with context compared to those without, *M_d_* = 0.66, *t*(23) = 4.20, *p* < 0.001, *d* = 0.80. Finally, a dependent samples *t*-test showed that contextualized indeterminate sentences were rated similarly to full-VP sentences, *M_d_* = 0.08, *t*(23) = 0.34, *p* = 0.741, *d* = 0.12.

**Table 2:** Means (SD) for the fill-in-the-blank and plausibility ratings tasks in Experiment 1.

Task	No context	Context
Fill-in-the-blank^a^	0.59 (0.24)	0.81 (0.22)
Plausibility ratings		
Indeterminate	3.60 (1.00)	4.26 (0.76)
Full VP control	-	4.35 (0.89)

a Proportion of participants that generated the dominant verb.

Collectively, these results show that contexts contribute to the interpretation of indeterminate sentences by (a) constraining the range of event interpretations and (b) enhancing plausibility. It should be noted that our sentences, which used proper names, were less constraining than standard experimental sentences, which typically use semantically rich agentive nouns such as *author* or *student*. Similar to neutral condition of Lapata et al. (2003), we also aimed to determine the effect of verb-noun relations without the added semantic constrain of the agent. The data seem to suggest a preference for a particular meaning for the filing event – which in our case was more often associated with the *agentive* role.

The fill-in-the-blank convergence rates and plausibility ratings in the no-context condition were likely lower here than what has been observed in other studies (McElree et al., 2001; Traxler et al., 2002; Pickering et al., 2005). But our results nevertheless support the principle that extra sentential context is frequently required to enhance acceptability of indeterminate VPs, particularly when event information is insufficiently constrained by the intra-sentential semantics. We suggest, moreover, that this is often the case when indeterminate sentences are presented in isolation, even when the sentence has a semantically rich agent, as in *The carpenter began the table*. Although *carpenter* and *table* alone could yield a range of event representations (e.g., *building*, *demolishing*, *sanding*, *varnishing*, *repairing*, *renovating*, *restoring*, *measuring*, *moving*, etc.), such sentences likely breed uncertainty about the specific events that they convey when no information is available in the utterance context to constrain interpretations. The possibility that these sentences remain indeterminate can be further demonstrated by data of Lapata et al.: even with a typically *agentive* subject noun (e.g., *The author began…the book*) subjects complete sentences with verbs that are *not* in line with the agentive role (supposedly *writing*) in 47% of the cases. A similar pattern is obtained when the subject noun “favors” a *telic* interpretation (e.g., *The student began…the book*): although half of the sentence completions are in line with the telic role (supposedly *reading*), in 51% of the cases they are not.

The results of Experiment 1 – in conjunction with other studies reporting poor comprehension metrics of isolated sentences – underscore a crucial gap in the indeterminate sentence processing literature: event-enriching inferences have never been empirically demonstrated. To date, psycholinguistic experiments on indeterminate sentences have focused almost exclusively on time-course-of-processing paradigms using decontextualized sentences (but see de Almeida, 2004 and Traxler et al., 2005). These experiments typically show that indeterminate sentences produce online processing delays relative to a variety of control sentences. But while there is ample evidence that decontextualized indeterminate sentences are costly to process, we know little about the interpretations that these sentences generate. In the absence of direct empirical evidence that indeterminate sentences trigger event-enriching inferences, it is prudent to assume the null hypothesis – that the initial representations that individuals assign to these sentences are as indeterminate as the input. Experiment 1 suggested that, without context, it is unlikely that individuals consistently assign a specific event meaning to indeterminate sentences. The goal of Experiment 2 is to investigate (a) whether or not the proposition that listeners build of indeterminate sentences is indeed enriched over time, given sufficient contextual support, and (b) what is held in memory about the original indeterminate sentence.

## Experiment 2

Numerous experiments investigating long-term retention of linguistic stimuli have demonstrated that delayed recognition reflects the meaning that individuals assign to utterances. For instance, a variety of now classical studies on false recognition for sentences (Sachs, 1967, 1974; Bransford and Franks, 1971; Bransford et al., 1972; Johnson et al., 1973; Brewer, 1977) have shown that while individuals quickly forget the *verbatim* form of linguistic expressions, the underlying propositional content or “gist” is retained.

This phenomenon is observed during recognition tasks when individuals erroneously recognize an expression that is synonymous with a presented sentence, albeit structurally distinct. For instance, Sachs (1967, 1974) demonstrated that, when they hear or read sentences like (6a) embedded in long contexts, participants frequently falsely recognize a semantically unchanged foil sentence such as (6b) upon delayed testing (80 syllables or up to 27 s after original presentation). Critically, this error does not occur for foils like (6c) that convey a fundamental change in meaning. Sachs’ findings thus illustrated that sentences are transferred to LTM primarily in their semantic or propositional codes, which produces false recognition for altered sentences that convey the same proposition.

6.
  - *Target*: the founding fathers considered owning slaves to be immoral.
  - *Semantically unchanged foil*: owning slaves was considered to be immoral by the founding fathers.
  - *Semantically changed foil*: the founding fathers did not consider owning slaves to be immoral.

Later studies illustrated that the encoded “semantics” of a sentence is not restricted to its denotational representation. Information generated from pragmatic inferences are also encoded in LTM (Fillenbaum, 1971, 1974; Johnson et al., 1973; Brewer, 1977; Chan and McDermott, 2006). Moreover, sentences compatible with inferences that participants draw are often recognized more frequently than originally presented sentences (Johnson et al., 1973; Brewer, 1977), suggesting that information generated inferentially might ultimately supplant the denotational meaning of a sentence in LTM. For instance, participants who listened to a story about a boy who “was pounding a nail” later misrecognized a sentence that described the boy “using the hammer” more frequently than they recognized the original sentence, which made no mention of the instrument the boy was using (Johnson et al., 1973).

False recognition of inferred meaning has been extensively studied. And, typically, misrecognized inferences are either strongly implied (Johnson et al., 1973; Brewer, 1977; Chan and McDermott, 2006) or entailed (Bransford and Franks, 1971; Bransford et al., 1972; Rinck et al., 2001; Jahn, 2004) by the presented material. To our knowledge, this phenomenon has not been investigated in the context of sentence indeterminacy. We reasoned that if individuals indeed enrich *Lisa began the book* by ascribing the sentence a specific event meaning inferred from the utterance context, then they should misrecognize a fully determinate foil sentence such as *Lisa began reading the book* following a delay.

We used the contextual passages employed in Experiment 1 to investigate pragmatic event enrichment. Consider again the context presented in Table 1 when it includes the indeterminate sentence *Lisa began the book*. As illustrated in Experiment 1, the passage biases the interpretation that Lisa began reading the book, although there is no mention of the *reading* event. During a delayed testing period, we presented one of three probe sentences: the indeterminate sentence that was presented during acquisition, a biased foil sentence, or a non-biased foil sentence.

Whereas Experiment 1 showed that event-enriching inferences are not likely achieved without context, the present experiment investigates whether the broader context indeed generates inferences for the biased event leading to enriched interpretations. Specifically, following Sachs (1967, 1974), we hypothesized that upon delayed recognition testing – in the present case, 25 s downstream – participants would falsely recognize biased foils but correctly reject non-biased foils.

Our experiment also addressed a secondary question concerning the time-course of the enrichment process and the nature of the proposition held in memory – whether original or “gist.” Do enriched interpretations supplant the denotational representation of a sentence during acquisition or only during retrieval? We propose that the decision difficulty associated with long-term recognition responses might shed light on this question. Specifically, the longer it takes to misrecognize the foil, the more likely it is that the denotational representation of the sentence lingers, competing with the inferred event propositional content computed from context. In contrast, relatively rapid misrecognition would suggest that the event inference merged with or even replaced the denotational meaning of the sentence during acquisition, and only a single, fully enriched representation of the sentence was encoded.

### Method

#### Participants

Seventy-two Concordia University students participated in this study – none of them participated in Experiment 1. They were all native speakers of English, and were compensated with course credit for their participation in a 40-min experimental session. They all gave informed written consent. The experiment was approved by the Concordia University Human Research Ethics Committee.

#### Materials

The materials consisted of the same 24 passages of continuous discourse used in Experiment 1. Each passage was comprised of three sentences of biasing context followed by an indeterminate sentence, which in turn was followed by several sentences of neutral discourse (see Table 1). The indeterminate sentence always appeared as the second clause of the fourth sentence in the discourse.

For each of the 24 paragraphs, three recognition probe sentences were generated for testing, as seen in Table 1: the original indeterminate sentence, a foil sentence that was biased by the discourse context, and a foil sentence that was not biased by the discourse context. The verb for the biased foil represented the response generated by the greatest proportion of participants during the fill-in-the-blank task from Experiment 1.

A set of 24 filler passages, which did not conform to any of the experimental features described above, were also prepared and included in the set of materials. The filler passages were of a similar length to the experimental materials and were written in the same general style, also describing mundane events, but without including indeterminate sentences. All 24 experimental passages as well as the 24 filler passages were recorded by the same female, native speaker of English, using natural prosody.

#### Procedure

Participants were seated in front of an iMac computer running PsyScope X (Cohen et al., 1993) and were provided with noise-canceling headphones. The experimenter then read the following instructions, which were also displayed on the screen for the participants to read:

In the following task, you will be listening to a series of short stories through the headphones. At some point during playback, the story will be stopped and you will be presented with a few words on the screen. Your job is to indicate whether these words were present (exactly as they appear on screen) in the passage that you just heard. The words may form a full sentence, or just part of a sentence, but as long as they match word-for-word with a part of the passage you just heard, then you are to indicate a YES response. Otherwise, indicate a NO response. Use the keyboard in front of you to register your responses. Press the GREEN button if your answer is YES, and the RED button if your answer is NO.

Responses were registered on a keyboard with the “/” key marked by a green sticker for “yes” responses, and the “Z” key marked by a red sticker for “no” responses. RTs were recorded for each response. Participants were also instructed to rate how confident they were that their responses were correct on a 7-point scale, with 1 representing a guessed response and 7 representing total certainty. In addition to the “/” and “Z” keys, only the 1–7 keys and the space bar (for initiating trials) were visible on the keyboard.

During each trial, one sentence was presented for recognition at one of two probe points: immediate and delayed. The immediate condition occurred 0 s after the oral presentation of the indeterminate sentence, and the delayed condition occurred following an additional 25 s of neutral discourse (see Table 1). Each passage ran roughly 40 s long with the two probe periods occurring roughly 15 and 40 s after trial onset. To mask these probe points, testing of filler sentences occurred roughly 5 and 30 s after trial onset. The filler items were also used to balance the ratio of novel to repeated probe sentences throughout the experiment. While two-thirds of our experimental probes were novel (i.e., the biased and non-biased foils) and one-third was repeated (i.e., the indeterminate probe), the inverse was the case for the filler trials.

The session consisted of three practice trials, followed by the 24 experimental and 48 filler trials presented in random order. The item frames were distributed within six orthogonal lists, each containing four unique items for each condition – 3 (probe) × 2 (delay). Thus, each participant heard all of the 24 passages only once, and provided an equal number of responses in each of the six conditions. There were 12 participants per list.

#### Data Analyses

Recognition accuracy was used as the criterion in a mixed-effects logistic regression model, which tested for main effects of delay and probe type and all first order interaction terms. We conducted a secondary analysis on RTs to contrast the decision difficulty associated with the three probe sentences at the delayed test point. Given that the three sentences have different lengths, reading demands differ from probe to probe. Thus, we computed a variable to isolate RTs associated with decision difficulty alone. Specifically, we subtracted from each observation in the delayed condition the mean RT of the corresponding sentence in the immediate condition, the latter of which included only correct responses [e.g., RT_biased/delay_ – mean (RT_biased/immediate/correct_)]. These RTs were used to assess objectively the degree of difficulty associated with long-term recognition for each sentence type.

### Results and Discussion

**Figure 1:**
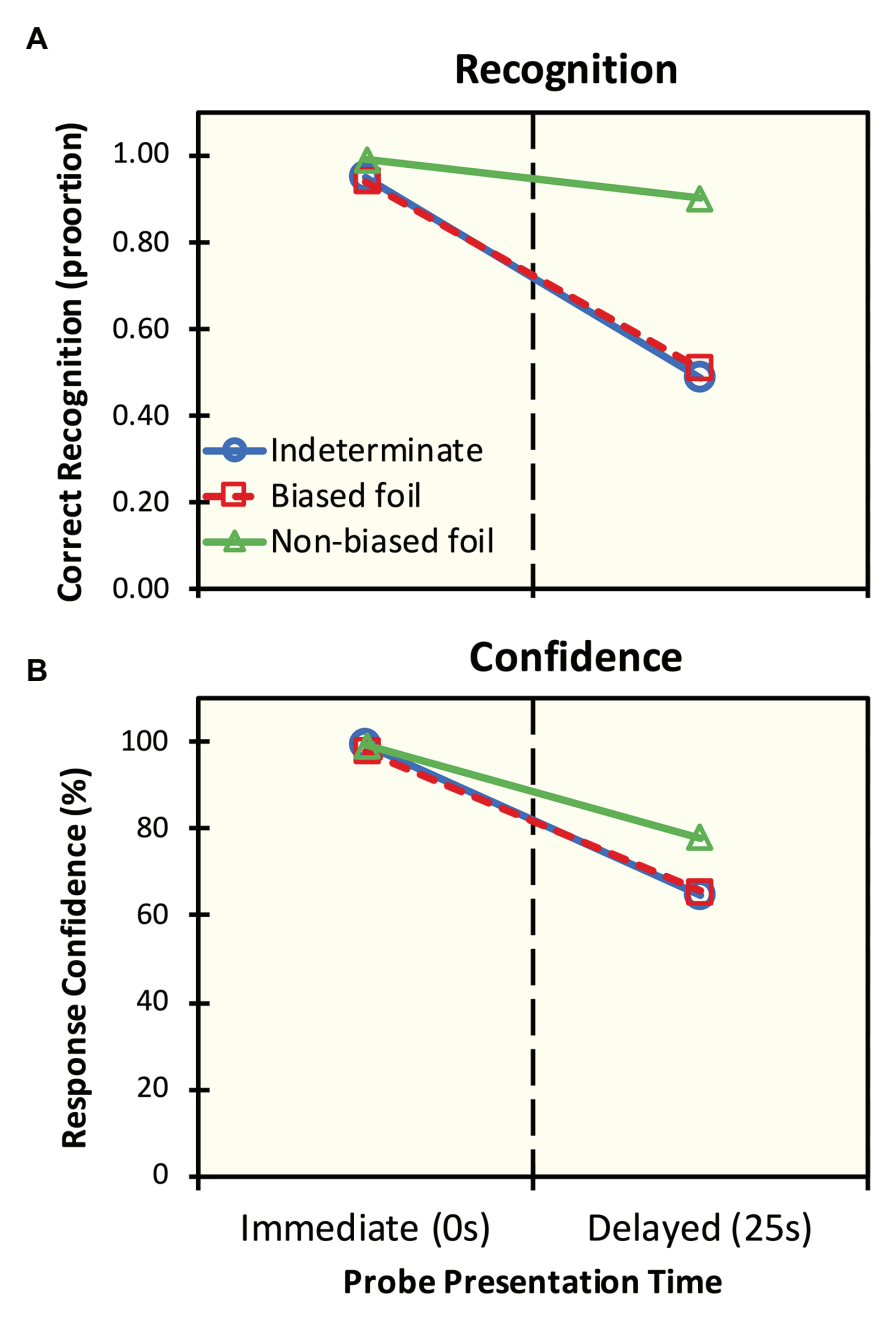
Results from Experiment 2. Recognition accuracy **(A)** and response confidence ratings **(B)** for visual probe sentences shown at the offset of the original auditory presentation (immediate) and after 25 s (delayed) of intervening neutral discourse for indeterminate sentences (e.g., *Lisa began the book*), biased foils (*Lisa began reading the book*), and non-biased foils (*Lisa began writing the book*).

One item was removed from the analysis due to a typographical error in the probe sentences that were presented for testing. Thus, all subsequent analyses were conducted with 23 items. Figure 1 presents recognition accuracy and confidence ratings. Descriptive statistics for these data are presented in Supplementary Material S3.

A binomial logit mixed model was fitted to the data using the “lme4” package (Bates et al., 2013) in R (R Development Core Team, 2012), with participants and items included as random effects, and delay period and probe type included as fixed effects. The overall model was evaluated relative to a null model consisting of only random predictors, and was found to provide a better fit to the data, *χ*^2^(5) = 483.75, *p* < 0.001.^4^ A summary of fixed effects is presented in Table 3.

**Table 3:** Logistic regression of recognition accuracy predicted by delay period and probe type in Experiment 2.

Predictor	Estimate	SE	*Z*	*p*	OR (e^E^)	95% CI
Constant	2.94	0.27	10.84	<0.001	18.87	[11.10, 32.09]
Delay (delayed)	−2.96	0.29	−10.14	<0.001	0.05	[0.03, 0.09]
Probe (biased)	0.07	0.37	−0.18	0.857	0.94	[0.45, 1.93]
Probe (non-biased)	2.07	0.76	2.73	0.006	7.91	[1.79, 34.87]
Interaction terms						
delayed × biased	0.14	0.41	0.35	0.730	1.15	[0.52, 2.55]
delayed × non-biased	0.03	0.79	0.04	0.967	1.03	[0.22, 4.85]

Our analysis revealed that recognition accuracy diminished with delay. In particular, the odds of correct recognition in the immediate testing period were 20 times that of the delayed testing period. An effect of probe type was also observed. The odds of correct recognition for the non-biased foils were 7.91 times that of the indeterminate probes, but the odds were approximately equal (*OR* = 1.15) for biased and indeterminate probes. Interactions were not statistically significant, as identical and biased foils patterned together in both delay conditions – i.e., at least 94% accuracy upon immediate testing and chance performance at delayed testing – and the non-biased foils differed from the others in both testing periods.

These results show that participants are able to differentiate the indeterminate sentences from the foils immediately following the presentation of the indeterminate sentence in the discourse with at least 94% accuracy and 98% confidence for all probe types. However, during delayed testing, participants incorrectly recognized the biased foil, but not the non-biased alternative, and response confidence was as high as 4.99/7 (67%) for trials in which recognition of biased foils was false.

Our results extend Sachs’ (1967, 1974) classical findings in significant ways. Sachs had shown that that “gist” representations serve as the primary source of sentence recognition in LTM once the verbatim trace has decayed. Recall that in Sachs’ studies, with the exception of the semantically anomalous condition, all probe sentences – identical and foils – conveyed virtually the same proposition. Beyond Sachs’ results, we show that the participants falsely recognize a probe that *does not* convey the proposition expressed by the original sentence presented in discourse: rather they accept sentences that are false, albeit contextually plausible. And participants do so with the same accuracy and confidence with which they accept the original sentence. Several other studies in recognition memory have pointed to the high acceptance and confidence associated with foils that are synonymous with the original stimulus sentence (e.g., Brewer and Sampaio, 2006). But, in the present case, *begin the book* and *begin reading the book* are not synonymous, for the former but not the latter is compatible with numerous events and both have different truth conditions.

In order to further investigate the processes underlying these false memories, we analyzed RTs associated with delayed recognition. Specifically, we measured the increase in RT from baseline by subtracting mean RTs for correct responses in the immediate condition from the RTs of the corresponding delayed condition. RTs that exceeded ±2.5 SDs from the mean – calculated separately for each condition – were replaced with the condition mean. This amounted to 2.6% of observations [24 indeterminate responses (16 at delay), 12 biased-foil responses (6 at delay), and 9 non-biased foil responses (5 at delay)].

**Figure 2:**
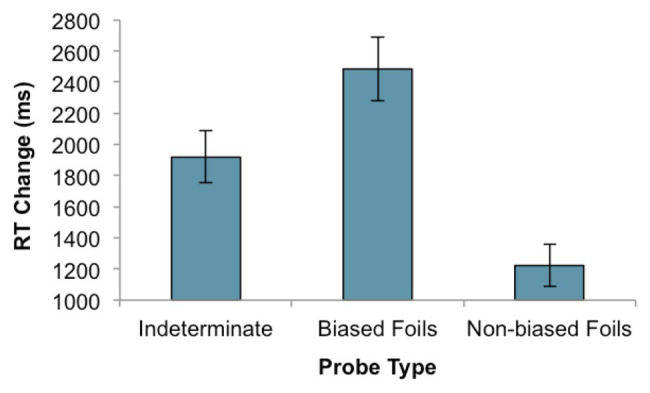
Mean change in response times from immediate to delayed recognition in Experiment 2. Error bars represent the SE, calculated with data averaged by participants.

A linear mixed-effects model was fitted to the RT data with probe type entered as a fixed effect and participants and items entered as random effects. The probe model was compared to a null model consisting of only random predictors and was found to provide a better fit to the data *χ*^2^(2) = 47.74, *p* < 0.001. Table 4 presents the results of the fitted model, in which we observed a statistically significant estimate for both the biased and non-biased foils compared to indeterminate sentences. Specifically, participants were faster to respond to non-biased foils, *d* = −0.54, ~95% CI [−0.79, −0.28] and slower to respond to biased foils, *d* = 0.36, ~95% CI [0.13, 0.59].^5^ Mean RT change is presented in Figure 2.

**Table 4:** Linear mixed-effects model of response time (RT) change by probe type in Experiment 2.

Predictor	Estimate	SE	*t*	95% CI (β)
Constant	1971	167.88	11.74	[1642, 2300]
Probe (biased)	458	168.49	2.72	[128, 788]
Probe (non-biased)	−712	168.36	−4.23	[−1042, −382]

Biased and non-biased foils both differed from indeterminate sentences in terms of being novel, and thus both required a negative response. They also both differed from indeterminate probes in terms of having one extra word – e.g., *reading/writing*. But neither of these differences can explain our results, because we removed such extraneous variance by computing a delayed-minus-immediate difference score within each probe condition. Any processing difficulty associated with reading or rejecting the foils was thus internally controlled. The residual decision time, which differed in each condition, suggests that different processes governed responses for each of the three sentences.

Semantic coherence with the content of the discourse appears to be the primary source of decision difficulty. While the non-biased foils, which contradicted the event schemas described in the passages, were easiest for participants to classify, indeterminate, and biased-foils both generated additional decision delays. Presumably, participants who inferred that the biased event – say, *reading* – occurred in the discourse had to decide whether or not they acquired this information from the originally presented sentence. But why should this decision take longer for biased foils compared to indeterminate probes, especially since the two engendered equal levels of accuracy and confidence? We propose that there are additional interferences associated with biased foils due to denotational remnants of the acquired sentence. In other words: the denotational representation of the indeterminate sentence presented in discourse during acquisition interferes with the contextually-favored biased foil when this is evaluated in the delayed recognition condition.

Few studies have explored RTs to understand the processes that govern false memories (Jou et al., 2004; Coane et al., 2007; Jou, 2008), and this literature – which is restricted to the Deese/Roediger-McDermott (DRM) paradigm – is consistent with our findings. Participants are slower to respond to strongly biased foils compared to both non-biased foils and true items (Coane, et al., 2007; see Lopes and Garcia, 2014, for review). This pattern could be understood in terms of an activation/monitoring process (Roediger et al., 2001), whereby the false information is initially activated by the presented material during acquisition and is later erroneously reconstructed during retrieval due to a source monitoring failure. This dual-process account suggests that true and false information are encoded differently and produce phenomenologically distinct memory traces. However, the effects we obtained are more in line with Fuzzy-Trace Theory (FTT; Reyna et al., 2016, for a recent review). This theory also postulates a dual-representation of semantic information – verbatim (i.e., identical) and gist (which is meaning-preserving but not identical to the original stimulus). We assume that responding to a verbatim probe (*Lisa began the book*) requires the retrieval of the original proposition, a decision that takes longer in the delayed probe point due to (a) the intervening neutral context, but primarily (b) the inferences computed from contextual information. Yet, longer RTs for the biased foil (*Lisa began reading the book*) suggest that an enriched proposition is also available. Together with data on recognition and confidence, our RT data suggest that dual-representations – original and enriched propositions – are formed in the course of memory encoding over time. Beyond FTT, results compatible with the idea that sentences can yield true and false propositions were obtained by studies investigating “garden-path” sentences such as *While Susan wrote the letter fell off the table* (Christianson et al., 2001). When presented with these sentences, participants respond “yes” about half the time to questions that suggest a misparsing analysis, such as *Did Susan write the letter?* While it is possible that the subject of the matrix clause (*the letter*) is a plausible (although implicit) object of the subordinate clause, these results suggest that both propositions, [*Susan wrote the letter*] and [*The letter fell off the table*], linger in memory.

Our study was not designed to investigate these theoretical alternatives but rather the specific phenomenon of indeterminate sentence enrichment. Nonetheless, if the dual-representation account applies to the present study, the pattern of RTs we observed would suggest that the event inferences drawn from context and from the denotational representations of indeterminate sentences were encoded as distinct memory traces in LTM: a true proposition and a false, contextually-enriched one. It is theoretically possible that an event inference and a denotational interpretation would have been encoded as a single enriched proposition. But had this occurred, delayed recognition responses for biased foils should have been on par with the indeterminate sentences. In the General Discussion, we elaborate on the implications of this finding for our understanding of the enrichment process.

## General Discussion

We conducted two experiments to investigate whether indeterminate sentences like *Lisa began the book* are enriched by assigning an interpretation that includes a specified event, such as *reading* in isolation and in context. Moreover, we were interested in the nature of the proposition that is obtained in memory for these sentences, both as participants first listen to the sentence and over time, as a function of context. In Experiment 1, participants were more likely to provide a dominant event interpretation when inferences were constrained by a broader discourse context. Similarly, participants provided higher plausibility ratings for indeterminate sentences in context, at levels comparable to event-specified sentences like *Lisa began reading the book*. Thus, Experiment 1 showed that, to the extent that indeterminate sentences generate event-enriching inferences, they are more likely to occur when interpretations are constrained by a broader discourse context. Lapata et al. (2003) have suggested that, in isolation, indeterminate sentences with neutral subjects (such as *Lisa*, in the present case) are filled primarily with a verb that is taken to be default for its object – such as *reading* for *began___the book*. In Experiment 1, we have shown that these “defaults” – if true – are ruled out by contextual demands. Compatible with classical studies demonstrating contextually-specific activation of properties in memory (e.g., Barsalou, 1983; McKoon and Ratcliff, 1988), our study shows that there are no defaults, but contextually appropriate enrichment.

Although contextual influence on sentence interpretation might be the norm, it should be noted that interpretations relying on some form of local semantic enrichment have long been assumed to occur, even for sentences in isolation. The GL framework (e.g., Pustejovsky, 1995, 2011), for instance, proposed a theory of coercion by which event meanings, such as *reading* and *writing*, are retrieved from the internal semantic representation of the nominal *book* and interpolated within the semantic representation (/logical form) of the sentence, as a kind of default. Thus, according to this view, a broader discourse context is not *necessary* for event-enriching interpretations to occur, for local semantic computations ought to determine the nature of the semantic filler, thus resolving the alleged mismatch between verb and complement.

To date, the vast majority of psycholinguistic and cognitive neuroscience experiments on indeterminate sentences have advanced theories of processing on the basis of sentences presented without context, de-emphasizing the role of context and even co-text in the event enrichment process, on the assumption that some sort of default meaning by necessity would ensue. Traxler et al. (2005, p. 5), for instance, proposed that “knowledge needed to enrich a complement is activated in an automatic and cost-free manner”… and that “the costs are due to additional operations needed to construct the appropriate event sense for the complement.” The building of “the appropriate event sense” according to these authors is in line with Pustejovsky’s interpolation proposal, whereby, say, *reading* is retrieved from *book*. This view, as we have argued elsewhere (de Almeida and Riven, 2012; de Almeida and Lepore, 2018) begs the question as to how the information that is “appropriate” is judged to be so. In order to enrich the propositional content that a sentence conveys (rather than to enrich a *sentence* qua linguistic object), there appear to be two alternatives: one is to rely on meaning decomposition, which in turn requires a criterion for determining what sort of content a concept carries (what is “analytic”) from the content that is contingent on one’s experience (“synthetic”). Meaning decomposition proposals cannot escape from the analytic-synthetic distinction and, thus far, a criterion for such distinction has not been set (see Fodor and Lepore, 2002; de Almeida and Riven, 2012; de Almeida, and Lepore, 2018; de Almeida and Antal, 2020; see also Quine, 1953). An alternative includes the semantic type-shifting of the complement NP, as discussed above. This view is committed to an ontology of semantic *types* for NPs, relying moreover on principles that adjust these types to fit the verbs’ requirements. The assumption is that NPs carry information about their possible types, with semantic principles being informed about their modes of combination with their host verbs. Yet another alternative – one that we favor – is to assume that the content that enriches a proposition comes from contextual clues among other sources (expectations, beliefs, and conventions).

Our Experiment 1 suggests that event-enriching inferences are unlikely to occur reliably – that is, by *default* – in the absence of a strongly constraining discourse. Moreover, Experiment 1 showed that the plausibility of these sentences is tied to the availability of inferential constraints outside the indeterminate VP. We elaborate on the implications of these findings in conjunction with the results of Experiment 2 below.

### Context and Enrichment

The results of Experiment 1 are particularly relevant because, to date, no studies have demonstrated that participants in fact generate event-enriching interpretations with or without context. The goal of Experiment 2 was to provide direct evidence of event inferences with contextualized sentences. After listening to short stories, which included indeterminate sentences like *Lisa began the book*, participants falsely recognized fully enriched foil sentences such as *Lisa began reading the book*. These foils included verbs that were implied by the discourse but never overtly mentioned. Although these event verbs were absent during acquisition, participants expressed 66% confidence that they indeed heard sentences like *Lisa began reading the book*, the same confidence they expressed for the original *Lisa began the book*. Thus, event inferences computed during acquisition left traces concerning the activity that was *began*, *finished*, or *continued* by the agent of the sentence. This is the first experiment to provide direct evidence that individuals build specified event representations, such as *reading*, *writing*, and *baking*, which they ultimately ascribe to such phrases as *started the book*, *continued the letter*, and *finished the cake*.

These results diverge from a previous experiment, which suggested that participants probably fail to generate event-specific interpretations for indeterminate sentences when presented in isolation (McElree et al., 2006b). Although our results are largely compatible with McElree et al. (as we discuss below), two critical differences in our methodology have enabled us to find evidence for the representations that have previously been undetected. Firstly, we employed a recognition paradigm (Sachs, 1967, 1974) that elicits the interpretations that individuals encode – the “gist” or, more technically, the *proposition* expressed by the sentence. Numerous studies of sentence and discourse memory (Sachs, 1967, 1974; Bransford and Franks, 1971; Bransford et al., 1972; Johnson et al., 1973; Brewer, 1977) show that what participants ultimately recognize reflects their understanding of the acquired information. Using a similar paradigm thus allowed us to assess directly participants’ interpretations of our sentences. In contrast, McElree et al. (2006b) used acceptability ratings and processing times, which are good subjective and objective measures of processing fluency, but are not sufficiently sensitive to reveal the nature of the proposition that was encoded. More critically, our experiment embedded indeterminate sentences within strongly constraining discourse contexts. The participants of McElree et al. (2006b) saw sentences like *The carpenter began the table* decontextualized, and rated such sentences as less acceptable than controls. However, as our Experiment 1 showed, indeterminate sentences alone are likely insufficient to generate systematic event inferences. Although such sentences are likely to yield representations compatible with different events, individuals reading the sentence without a broader discourse context possibly fail to compute one specific interpretation, which is consistent with results of Lapata et al. (2003).

The results of Experiments 1 and 2 suggest that a detailed event schema must be established in the utterance context to produce sentence-enriching inferences. And in the absence of such contexts, indeterminate sentences breed uncertainty about the events that they are intended to describe. The idea that indeterminate sentences in isolation create uncertainty is supported by a recent fMRI study (de Almeida et al., 2016) showing the engagement of diverse brain areas – beyond those involved in the interpretation of fully determinate sentences – in particular the temporal and inferior frontal lobes bilaterally, the anterior cingulate cortex, and the thalamus. Although functional-neuroanatomical data should be seen with caution – for they alone cannot be taken to choose between theoretical or processing alternatives – they provide yet more evidence for how indeterminate sentences are (attempted) to be resolved: rather than resorting to local, default semantic operations, the resolution processes might involve generating inferences compatible with the original propositional content.

### The Time Course of Enrichment

Having suggested that sentence-enriching inferences occur in the process of interpreting indeterminate sentences in context, a further issue for explication concerns the locus of enrichment. At what level of representation – or processing interface – does an inference enrich one’s understanding of an indeterminate sentence? In the present study, delayed recognition RTs suggest the occurrence of interference between the denotational representation of the sentence and an enriched form, based on inferences computed from the discourse. Research on recognition for critical foils in DRM lists (Coane et al., 2007) suggests that RTs follow an activation/monitoring pattern (Roediger et al., 2001): participants are slower to respond to strongly biased words than they are to respond to non-biased words and true words (Coane et al., 2007). The additional interference associated with foils marks the presence of competition between originally presented and inferentially generated information. Compatible with FTT (Reyna et al., 2016), however, we take the false recognition of biased foils to be determined not by underlying associations but by the computation of a semantic alternative, a contextually-driven enriched proposition (“gist”), which is built over time and comes to compete with the original proposition.

Our participants’ RTs followed this pattern. Recognition was slower for biased-foils than for indeterminate sentences and non-biased foils. Thus, it is likely that a proposition based on inferences about the event is ultimately encoded apart from the denotational representation of the sentence. An implication of this multiple trace account for indeterminate sentence processing is that enriched representations may not be built into the original propositional representation *per se*, but instead might occur beyond its composition. And interpretations of what a sentence means vs. what it implies are computed at distinct levels of representation – the former at the syntactic-semantic interface, and the latter at the level of thought, or pragmatics.

At first, our data can be seen as compatible with different accounts of indeterminate sentence processing, including those for which *interpolation* is a requisite for composing a semantic representation of the sentence. It may be that interpolation is automatically triggered by the input, and that this semantic operation works in tandem with context-driven inferential processes to produce a fully enriched sentence meaning. Specifically, it is possible that the context constraints the nature of events, providing information about a plausible predicate (e.g., *reading*) that serves to further enrich incoming sentences. When the sentence is parsed – and a mismatch is detected – the event suggested by the context becomes a predicate within the proposition encoded in memory. This process is compatible, then, with a full interpolating account of coercion, but one in which discourse information (viz., inferences based on implied events) provides the predicate for the local structural computations and yielding an enriched proposition. It is difficult, however, to determine how the content of a given context provides these directives – although it is possible to conceive of a general mechanism such as a “scoreboard” (Lewis, 1979) filled with common-ground information and presuppositions. And it is also difficult to constrain the boundaries of the context (see Cappelen and Lepore, 2005). However, while context *suggests*, it does not *determine* sentence enrichment. We note this because contextually supported foils are rejected immediately and only later – over time, at the second probe point – they are accepted with relatively high levels of confidence. This, to us, suggests that the process of enrichment is primarily – if not uniquely – contextually-driven. Moreover, our data also suggest that the original proposition lingers, for sentences compatible with the original content of encoding are also accepted with the same level of confidence as the foils. If there is default coercion, the original, unenriched propositions should not linger in memory. Also, RTs to original and contextually-supported foils differ significantly, in the contrast between probe points, with greater costs for the enriched sentence at the late probe point. This suggests that, although seemingly confident, participants are reluctant to accept the enriched sentence, even when it is consistent with – and perhaps highly suggested by – the context. Therefore, while in principle compatible with the interpolation view, we see our data suggesting a process of enrichment that is primarily – if not uniquely – determined by contextual information over time.

Yet, another reason for casting doubt on the interpolation alternative relies on the theoretical morass that a commitment to analyticity entails (see, e.g., Quine, 1953; Fodor and Lepore, 2002; de Almeida and Antal, 2020). Simply put, there are no firm criteria for distinguishing between properties that are *constituents* of a concept (e.g., what goes into the “qualia structure”) from those that are not. While this argument is not central to the interpretation of our results, it is a challenge to a proposal that relies on definitional or contingent properties of objects and events as contributing information to semantic computations.

What our experiments do not rule out is that sentences such as *Lisa began the book* might be, at first, subject to semantic algorithms that compute *semantic types*, thus triggering type-shifting operations akin to Partee (1986) and further extensions of type theory (e.g., Asher, 2015), without postulating lexical-semantic interpolation. These formal operations can very well precede interpretations (i.e., logical forms) that later become further enriched by context. Alternatively, it may be that pragmatic inferences are built on denotational representations of sentences derived from classical compositional mechanisms built out of unenriched syntactic analyses (de Almeida and Dwivedi, 2008; de Almeida and Riven, 2012). Either way, it is clear from our results that pragmatic inferences indeed occur in the service of enriching indeterminate sentences, playing a crucial role in building enriched propositions, thus in part accounting for effects obtained with behavioral and neuroimaging techniques.

## Conclusion

Indeterminate sentences presented within strongly biasing discourse contexts trigger event inferences, which are encoded in LTM and later falsely recognized. Both the recognition of contextually biased sentences in the delayed probe point together with their longer RTs most likely suggest a competition between the original, *unenriched* sentence proposition and the proposition enriched with inferences computed from context. Our results are compatible with studies showing high rates of acceptance of false probes that are synonymous with original sentences (e.g., Sachs, 1967, 1974; Brewer and Sampaio, 2006) or that are their logical and pragmatic implications (Brewer, 1977). In the present study, however, the false-memory effects we obtained were even more surprising because they did not involve synonymous or entailed sentences. Taken together, the results from both experiments suggest that enrichment and consequent false recognition of indeterminate sentences can be attributed primarily to information generated by the context rather than to a default semantic interpolation.

More broadly, our study contributes to understanding the investigation of the division of labor between semantics and pragmatics, and their computations in the course of language comprehension. The cases we mentioned in the opening paragraph of the present article – viz., of “unarticulated constituents” (Perry, 1986; Recanati, 2004) – are examples of a pervasive approach to sentence meaning, namely one that takes a linguistically unmotivated form of silent meaning to contribute content to the representation of a sentence, beyond what it explicitly says. We suggest that sentences might hold their compositional meanings – without default interpolation – in isolation, with context being the source of enrichment in the form of pragmatic inferences computed over time.

## Data Availability Statement

The raw data supporting the conclusions of this article will be made available by the authors, without undue reservation.

## Ethics Statement

The studies involving human participants were reviewed and approved by the Concordia University Human Research Ethics Committee. The patients/participants provided their written informed consent to participate in this study.

## Author Contributions

RdA devised the study. LR and RdA designed the experiments and wrote the manuscript. LR prepared materials, conducted the experiments, and analyzed the data. RdA is the main responsible for the final manuscript. All authors contributed to the article and approved the submitted version.

### Conflict of Interest

The authors declare that the research was conducted in the absence of any commercial or financial relationships that could be construed as a potential conflict of interest.

## Footnotes

^1^We use *indeterminate* because, as we have noted (de Almeida and Riven, 2012), the actual event to which these sentences refer is not overtly specified, making sentence (1) compatible with any event over which *begin* would have scope. We eschew the terms *type-shifting* or *coercion*, as employed in the literature, because they are committed to particular theories on how indeterminate sentences are enriched – namely, by *type-shifting* or *type-coercing* the complement nominal (e.g., Pustejovsky, 1995, 2011; Pylkkanen, 2008; Asher, 2015), as we briefly review below. The use of the term *indeterminate,* in this regard, is *theory-neutral* with respect to whether or not sentences such as (1) are enriched, and, thus, more in line with the null hypothesis. We also want to avoid confusion with other terms, such as *underspecification*, which have been used in the semantics literature to mean something different from what we claim is indeterminacy. In some circles (see, e.g., Frisson, 2009), underspecification is associated with a representation based on features, some of which are absent in certain uses of a word, on the assumption that words are polysemous and represented as sets of features. We are not committed to features as constituents of lexical meaning, which we see as an empirical and theoretical issue yet to be determined (see also Fodor, 1998).

^2^It is also noteworthy that much of the research reviewed here has focused on a class of verbs that may have two subtypes: aspectual verbs (e.g., *begin*, *finish, continue*, etc.) and psychological or “try” verbs (e.g., *enjoy*, *prefer, attempt,* etc.; see de Almeida and Dwivedi, 2008). Katsika et al. (2012) suggested that these subtypes are processed differently. Although most papers in our review focus on a superordinate class that includes both subtypes, our discussion and, more importantly, the present experiments focus exclusively on the aspectual subtype, which is thought to be more exemplary of the processing effects reported in the literature.

^3^The preferred/non-preferred classification was based on the most frequently given verb in frames like *The secretary was _____ the memo* from McElree et al. (2001). Verbs used in the preferred condition were provided more frequently (66%) than verbs used in the non-preferred condition (7%).

^4^Value of *p* less than 0.000 were reported as *p* < 0.001. Otherwise, the exact value of *p* is reported.

^5^Effect size estimates (*d*) and approximate confidence intervals for *d* reported in text, as well as the condition means and SEs presented in Figure 2 were all calculated with data averaged by participants, unlike the mixed-effects model in which all unique observations were included.
