# Supplementary material for: Context Breeds False Memories for Indeterminate Sentences
Source: Front Psychol. 2021 Mar 12;12:616065. doi: 10.3389/fpsyg.2021.616065 (PMC7994259; doi:10.3389/fpsyg.2021.616065)
Supplement: Supplementary file 1 [file Data_Sheet_1.pdf]

frontiers

Supplementary Material S1

Context Breeds False Memories for Indeterminate Sentences

Levi Riven[1] & Roberto G. de Almeida[1*]

*** Correspondence:**
Roberto G. de Almeida
roberto.dealmeida@concordia.ca

Instructions for Experiment 1:

Fill-in-the-blank task

“Please complete each sentence below by filling in the blank space (column B) with a verb. A verb is any word depicting an action or mental state, such as jump, wait, or imagine. Feel free to use the past or present tense as long as it completes the sentence in a grammatical manner. Thus, the sentence "The children were____on the ice rink" should be completed with "skating" and not "skate" or "skated." Also, complete each sentence with the verb that best fits the context of the sentence. For example, "The secretary____the phone" is best completed with "answered" as opposed to "invented" even though both responses are perfectly grammatical. Perhaps the best way to accomplish this is to respond as quickly as possible, relying more on instinct than creativity. Infinitive forms such as "to eat," "to bark" are also acceptable as supplements for "eating," "barking."”

Rating task

“This task requires that you read a series of short paragraphs describing a variety of mundane scenarios. Each description will end with a sentence (in italics) that you will be asked to evaluate on a scale from 1 to 5. Assign a low rating (1) to sentences that seem to fit poorly with the paragraph or that sound awkward to you in some way. Assign a high rating (5) to sentences that flow well with the overall scenario and that seem well phrased. For intermediary sentences, use ratings 2-4. Note however, that we are NOT asking you to evaluate the quality of the writing per se, but the suitability of the closing sentence given the context. Thus, sentences that seem unusual should get low ratings, but sentences that are consistent with everyday English and follow naturally from the paragraph should get high ratings (even if they are unworthy of the Pulitzer Prize).”
