# Supplementary material for: Context Breeds False Memories for Indeterminate Sentences
Source: Front Psychol. 2021 Mar 12;12:616065. doi: 10.3389/fpsyg.2021.616065 (PMC7994259; doi:10.3389/fpsyg.2021.616065)
Supplement: Supplementary file 2 [file Data_Sheet_2.pdf]

Supplementary Material S2

Context Breeds False Memories for Indeterminate Sentences

Levi Riven[1] & Roberto G. de Almeida[1*]

*** Correspondence:**
Roberto G. de Almeida
roberto.dealmeida@concordia.ca

Experimental Materials

Below are the 24 experimental passages used in our study. Within each passage, the indeterminate sentence appears in italics, and '|' marks the beginning of the neutral discourse. The three recognition probes are presented after each passage as follows: *Indeterminate/biased foil/non-biased foil.*

(1)

Lisa had been looking forward to the new Grisham novel ever since it came out. She had finally managed to set aside some time this weekend and made sure to make her home library nice and cozy. First thing Saturday morning, Lisa curled up on the sofa in her library with a blanket and a fresh cup of coffee. With everything in place, *Lisa began the book.* | Suddenly, the doorbell rang. Lisa grunted, put down her coffee and sluggishly made her way to the kitchen. It was her neighbour John and he was out of peanut butter again. Looking through the cupboard, Lisa realized she was no better off. She told John he was out of luck and suggested he try calling Marie, their mutual neighbour.

Recognition probes: *Lisa began the book/began reading the book/began writing the book.*

(2)

Dan was so busy with clients all day that he skipped lunch without even noticing. Now that his work was finally over, Dan was feeling hungry as he made his way home. When he arrived at the house, he dropped his things in the entrance, dashed for the kitchen, and scavenged the fridge for yesterday's leftovers. Finally in front of the TV, *Dan started supper.* | He flipped through the channels and was glad to discover a documentary on the American civil war. Dan had always been interested in U.S. history and he considered the civil war to be among the country's most dramatic tales. He had seen this particular documentary before and was impressed with the level of detail with which the story was told. He tried to remember the other documentaries that had been made by the same journalist. He made a mental note to look it up later.

Recognition probes: *Dan started supper / started eating supper/ started ordering supper.*

(3)

Claire had fallen behind this semester and was worried that she would not have time to work on her final project before the deadline. After all, a 15-page review of the history of the mind-body problem was an exhausting task. Fortunately, she had completed all of her research and had even drafted a detailed outline and introduction. Working diligently all night, *Claire finished the paper.* | When she awoke the next morning there was a message on her phone from her boss. A co-worker called in sick and they needed a replacement for the night shift. Claire knew she could use the money so she decided to put off her other plans for the night. She called her boss to say she would be glad to cover the night shift. Her boss told her she appreciated the offer, but one of her coworkers called just before she did. Claire said she didn't mind, but would like to increase her work hours if possible.

Recognition probes: *Clare finished the paper/finished writing the paper/finished recycling the paper.*

(4)

Ever since Edward published his first novel entitled The Meek Shall Inherit the Earth, his agent had been harassing him to produce more journalistic essays. He was juggling three projects at the same time, including a piece for the The New Yorker, which was due on Monday. But with his schedule entirely tied up in promoting the book, Edward was forced to dedicate his weekend to the New Yorker project. First thing Saturday morning, *Edward began the article.* | The radio announcer in the background summarized the day's stories in his dry yet chipper tone. One in particular caught Edward's ear. The New York Public Zoo had a break in the night before, and several animals were missing. Police initially suspected this was simple vandalism but further investigation revealed several smaller animals were missing. The story was still developing but strange reports coming from Central Park seemed to indicate that the critters had been released instead of stolen.

Recognition probes: *Edward began the article/began writing the article/began reading the article.*

(5)

Alison was really excited about her Canadian Idol audition. She would be performing "Amazing Grace" because she felt that the melody complimented her voice really well. When she was finally called in to the audition room, Alison entered the room, carefully approached her marker and introduced herself. Feeling the butterflies in the pit of her stomach, *Alison started the song.* | She kept an image of her mother in her mind. Her mother had been the most supportive person she knew. The past year had been particularly difficult, and her mother made sure to call her every week to check up on her. Alison had just moved to a new city to pursue her degree and was adjusting much slower than she had anticipated. Her mother kept her up to date on all the nieces and nephews to give her a feel of home. Alison found a job right away but was finding it a particularly demanding position and was having trouble balancing work and school. Her mother told her to sort out her priorities and find a different job if she had to.

Recognition probes: *Alison started the song/started singing the song/started playing the song.*

(6)

Irene was in for a twenty-six-mile marathon today and she was well prepared. She had gradually increased her endurance on the treadmill over the past year and was now able to go for six hours uninterrupted. On the day of the race, Irene got off to a strong start conquering the first fifteen miles

in just over two hours. And in only 4 hours, *Irene completed the track*. | The celebrations that followed were momentous. All her family and friends had gotten together to throw her a surprise party at her place, and they thought it would be grand to invite everyone she knew. It had been a long while since she had been out with her friends, what with her jammed-packed schedule. Irene was one of those people who handled stress effortlessly and could not tolerate boredom. She was always most comfortable in the thick of things, and her choice of work reflected that admirably.

Recognition probes: *Irene completed the track/completed running the track/completed paving the track.*

(7)

Carlos had fallen behind in his research this semester due to the heavy course-load. He was teaching three classes for the first time in his academic career and was feeling the pressure of the job. He had just returned the midterm exams to all of his students, and he regretted promising them that he would return the papers so soon. Clearing his schedule for the weekend, *Carlos finished the assignments*. | Late Sunday evening, he noticed a message on his phone. It was the department secretary and she said it was rather urgent. He called her back immediately to determine what the issue was. Carlos encountered problems with the secretary before and had a very low tolerance for bureaucratic mishaps. She told him that the organizing committee for a new field conference had contacted the university hoping to reach him. Carlos was torn between excitement and dread. This conference was a fantastic opportunity for his research agenda, but he couldn't imagine how he could possibly take on the added responsibility. He rubbed his eyes and shook his head. At least this was one of those good problems.

Recognition Probes: *Carlos finished the assignments/finished grading the assignments/finished writing the assignments.*

(8)

Every Wednesday, Tamara entertained her kindergarten students by recounting a classic Disney adventure in her own words. She was midway through Beauty and the Beast when a bluebird flew into the classroom. A rare sight in an urban neighbourhood, the children's interest in the tale suddenly transferred to the present fascination. As soon as the children's curiosity wore off, *Tamara continued the story*. | Just then the principle's voice came on the loud speaker. She was announcing a routine fire drill that would take place in the next class period. The children started to buzz with excited chatter. To them, a fire drill was practically an extra recess. It was not class, and it was not indoors, and that was good enough. Tamara was a little annoyed at the number of interruptions today but decided that a nice break of sunshine was just what she could use at the moment. As the class period stretched on, the children were braced with anticipation.

Recognition Probes: *Tamara continued the story/continued telling the story/continued writing the story.*

(9)

Damian Biggs is the front man for the 3 piece punk band Road Rage. Since the early 90's, the band has been the darlings of the local punk scene as droves of angst-ridden teenagers swarmed the stage at The Dungeon to see Damian and co take on "The Man." Surprisingly, the band has aged well,

headlining a sold out concert last night at The Arena. The 20,000 spectators stood in silent anticipation as *Damian started the guitar*. | This was the first appearance for Road Rage in just over a decade. Damian wondered why they had not got the gang back together sooner, though the drummer Mitch was not feeling quite as reminiscent as Damian. The two had been friends since the days of skipping out on high school classes, bumming smokes from the most jaded of teachers after class. Road Rage was born out of a couple of garage-sale guitars and the inevitable boredom of Sunday afternoons. In a way Mitch was still holding on to that image of the band, though he couldn't bring himself to tell Damian as much after all this time.

Recognition probes: *Damian started the guitar/started playing the guitar/started listening to the guitar.*

(10)

Pam was enjoying her lunch that Alan had prepared for her this morning. But about halfway through her chicken sub, her boss called her into his office. This wasn't the first occasion her lunch was interrupted for some triviality, and so Pam took the liberty of bringing her food with her. Once inside his office, *Pam continued the sandwich*. | Of course, it was a trivial matter that "needed" consulting. Her boss was flipping through carpet samples and wanted Pam's opinion. Helping her boss match carpet swatches with paint samples was not exactly what Pam had in mind for this job. She thought of these encounters, occurring several times a week, to be a test of sorts, of her patience more than her capacity for interior decorating. As her boss prattled on about the pros and cons of her two favourites, Pam began to wonder what her boss actually did here besides plan her home renovations.

Recognition probes: *Pam continued the sandwich/continued eating the sandwich/continued dressing the sandwich.*

(11)

Paul was becoming increasingly confused with the material for his PSYC 380 class. His instructor was extremely knowledgeable but perhaps was too bright to communicate the material to an undergraduate audience. During last week's lecture, Paul had begun inquiring about a particularly evasive concept just before the class ended. So at the outset of today's lecture, *Paul continued the question*. | When the sound of the final bell came it was almost a comfort, even though Paul was left unsatisfied yet again. He grabbed his knapsack from beneath the seat and filed out of class with the rest of the students. Everyone rushed out to the front exit and bottlenecked through the doors to the bus stop. While the mob of students chattered and buzzed with the excitement of an ended school week, Paul was lost in the pages of the latest addition to his collection. For a year he had been under the spell of a little-known novella author out of Nebraska. How the man even got published was part of the mystery.

Recognition Probes: *Paul continued the question/continued asking the question/continued answering the question.*

(12)

Sebastian was commissioned by The Museum of Canadian History for an exhibit on the Canadian Prime Ministers. Sebastian was the artistic director of the exhibit and his job was to create large-scale

depictions of the Prime Ministers. Sebastian rented a studio loft near Rideau Canal and got to work on Sir John A. Macdonald. After several long sessions, *Sebastian finished the portrait.* | His knuckles were stiff. He hadn't realized how cold it had become inside. He hopped down off the stepladder and snatched a sweater off the back of the closest chair. A cool draft was picking up from the bay windows facing the canal. Sebastian hadn't noticed that the light streaming onto the floor had paled and darkened. The draft was becoming loud and blustery, surging up against the windows and filling the loft with an irregular rattling. Sebastian did as best he could to cover the lower windows with some blankets and quilts lying around the couches.

Recognition probes: *Sebastian finished the portrait/finished painting the portrait/finished appraising the portrait.*

(13)

Jane was having trouble dealing with her alcohol addiction. She had gone clean for six months, attending AA meetings regularly, but recent stressors had thrown Jane off course. This morning, Jane didn't even get out of bed before turning to the whiskey stored at her bedside.

And in just a matter of seconds, *Jane finished the bottle*. | But no matter what, Jane couldn't get her mind off her divorce and losing the custody battle. The economic recession was an all too present concern, costing her half her hours at work. But these worries were subordinate to her concerns for Jeannie, the five year old daughter. Everything she did now was an effort at marketing herself as the better parent, but the odds felt stacked against her. In a moment of clarity, she realized that she needed to change a great deal if she wanted to win custody of Jeannie.

Recognition probes: *Jane finished the bottle/finished drinking the bottle/finished filling the bottle.*

(14)

It had been 3 long months of renovations and now the house was finally ready to be lived in. But a few small jobs still needed attention. Alan had promised Sally a new bookcase for her home office and he already collected the wood that he needed for the job. Just a day before moving in, *Alan completed the shelves*. | The house was much improved since before the renovations and Alan and Sally were eager to decorate. They both had their own pet projects picked out, though they thought it only fair to allow some room for each other's "consultations". Alan declared his domain in the kitchen, and the plans were beginning to coalesce in his head, from spice racks to salad tongs. Sally knew that the gift for cuisine was somewhat more apparent in Alan than herself, so she mostly deferred to his better judgment on these matters.

Recognition probes: *Alan completed the shelves/completed building the shelves/completed dusting the shelves.*

(15)

Staring at her rough draft on her computer screen, Sandra sighed. She was satisfied with the font that she had selected but was a little less than pleased with the phrasing. Sandra and Tom still hadn't decided on a caterer and the unfinished text before her was only making her feel more anxious. Taking a deep breath, *Sandra continued the invitation note*. | Next on her to-do list was the reception seating arrangements and the menu selections followed that. Suddenly, Sandra remembered that she

still had to contact the band they had finally settled on. This was not what she had in mind for her grand day. Well the day itself was going to be grand indeed, but she did not expect that she would be bearing the burden of the whole thing. Sandra pushed her chair out from the cluttered table, and sat very quietly for a while. She turned her gaze to the phone and thought of Tom.

Recognition probes: *Sandra continued the invitation note/continued writing the invitation note/continued sending the invitation note.*

(16)

Tammy was ready to deal with the mysterious odor that was emanating from the kitchen. Because she could not identify the exact source of the smell, she decided to scrub the kitchen from top to bottom. She had already emptied the kitchen of every last trace of food and was ready to get rid of the grime. Getting on her hands and knees, *Tammy completed the fridge*. |

Just then she noticed that her mail had fallen off the counter. She made her way over to lift the envelopes off the floor. Leafing through the various items, Tammy felt overwhelmed. Creditor after creditor was after her. She had been living above her means for a while without realizing how deep in debt she had got herself. A few months earlier, Tammy had a shopping frenzy and financed a car, an LCD television and a new washer and dryer. To boot, she had accumulated thousands of dollars in credit card debt. With her monthly expenses far exceeding her income and interest rates as high as 20%, Tammy came to terms with reality. She would be forced to declare bankruptcy.

Recognition probes: *Tammy completed the fridge/completed cleaning the fridge/completed stocking the fridge.*

(17)

Grace was excited about the prom because she had been putting together her own outfit from scratch. Her mom was a seamstress and after learning a few tricks, Grace felt like a natural with a needle and some fabric. Immediately after class, Grace rushed to her mom's workstation in their basement. With her foot on the pedal and a few pins pursed between her lips, *Grace continued the dress*. | When the phone rang on her bedside table, she dragged herself away. Following her mumbled hello, her friend Kylie began babbling on at a mile a minute on the other end of the line. Kylie had her own beau to bring to the ball, another rising football star named Chad. Lately they had been chatting daily about their dates, or rather Grace listened daily to Kylie go on about this latest crush. The phone calls used to be less regular, but they had a lot of different periods at school this year compared to years previous. Grace was glad to oblige her friend.

Recognition probes: *Grace continued the dress/continued sewing the dress/continued washing the dress.*

(18)

Justine was in the process of installing the new operating system on her computer when Mr. Sellers interrupted her with an urgent request. Many of the expense reports were being filed incorrectly lately and this was interfering with the processing of accounts. Mr. Sellers wanted Justine to take over a note he was working on that addressed this issue.

Picking up where Mr. Sellers left off, *Justine completed the memo*. | Once she got back to her own desk, she sat down and took a minute to look out the window and sip from her coffee. Having a window in her office was certainly a perk, but having one of the few trees on the lot just outside it was an added bonus. Justine had lost count of the hours spent doing “faux-work", collecting her thoughts for whatever projects she had going.

Recognition probes: *Justine completed the memo/completed writing the memo/completed reading the memo.*

(19)

Lena dreaded requests for letters of recommendations from her employees, but she was happy to do it for Ellen who was a motivated worker. Lena asked Ellen for two-week’s notice because articulating a person’s qualities could be a long and arduous process. Lena was just a few sentences into the task, when she was interrupted by a phone call from an important client.

Once she hung-up, *Lena continued the letter*. | Before long, the phone rang again. Lena frowned and muttered for a moment, expecting it to be the same client calling back for yet another clarification of some trivial minutiae. She braced herself before picking up the phone and put on her most placating tone. She was pleasantly surprised to hear her mother Judy on the other end, but she soon recognized the stress in her voice. She asked Lena if she was doing all right. Lena replied that she was quite fine and asked her mother the same.

Recognition probes: *Lena continued the letter/continued writing the letter/continued reading the letter.*

(20)

Amy wanted to truly satisfy Ted’s belly this Valentines Day. After all, "the way to a man’s heart is through his stomach," and if done right, a homemade meal can be very romantic. Amy knew that Ted loved poultry, especially when served with a festive flair like Thanksgiving and Christmas dinners. Placing all the ingredients in the oven, *Amy started the turkey*. | It was only 3 months ago that the two nearly split up. Ted was in the midst of brokering a new deal and he was spending long hours at work. Amy too had an increased workload because Ted was rarely home to help out with the children. They were both terribly stressed and would often butt heads when Ted finally did come home from work. To avoid the inevitable confrontations with Amy, Ted began frequenting McGovern’s Pub after work while Amy was left at home rearing the two boys.
Recognition probes: *Amy started the turkey/started cooking the turkey/started eating the turkey.*

(21)

The bakery was a mess and Judy still had many pastries to make for the next day, not to mention the usual selection of bread for the retail counter. At the top of her list was an order for Camden Elementary, the school her daughter attended. It was Katie’s birthday and Judy planned to sneak a birthday surprise into the school’s usual bread order. Finally at 11:00pm, *Judy finished the cake*. | At last, she could begin cleaning. It would be 2 in the morning before she would arrive home and Judy was dreading the inevitable fight with Tom that would follow the next day. Tom was emasculated enough that Judy was the sole breadwinner of their family. It only exaggerated Tom’s sense of insecurity when Judy came home late after a night of hard work. He would often get suspicious and make wild accusations about Judy’s fidelity. Judy always understood that these outbursts were never

really about her loyalty as a lover. The thought scared her, because it was apparent that her life's passion – her work – would always be conceived as a sort of betrayal.
Recognition probes: *Judy finished the cake/finished baking the cake/ finished eating the cake.*

(22)

Vera and Matilda met on the veranda at 4:00PM for snacks and gossip. Matilda had much information to divulge and she enjoyed delivering her news between sips of hot tea for dramatic effect. At four o'clock sharp, the maid brought the refreshments to the ladies waiting silently in a pair of lawn chairs. Before any exchange of information, *the ladies finished the biscuits*. | Vera was eager to hear the news of the affair and Matilda was happy to oblige between appropriate outbursts of mild outrage and disbelief. Of course it was no secret that Beatrice had been growing steadily unhappy this past year. Her husband had been taking on quite a few extra hours at the firm to keep up with the mortgage. The children were both away at schools out of province. The affair was really inevitable, Vera and Matilda agreed. Vera thought it was a wonder that it had not happened sooner. Matilda added that it was at least fortunate that Beatrice's husband hadn't found out about it yet.

Recognition probes: *The ladies finished the biscuits/finished eating the biscuits/finished baking the biscuits.*

(23)

Josh was a freelance computer programmer and he had just been hired for a job at Technology Software Solutions Inc. The company was looking to advance their R&D division and they needed someone with experience in various domains. Josh was responsible for a new inventory-tracker program that would be compatible with Blackberry as well as other hand-held devices. Eager to impress his new boss, *Josh started the code*. | Josh had only been at the company for a month and was eager for a chance to show off. No more than three minutes had passed when the competition poked his head over the cubicle wall. Josh always thought of Larry as the office troll, and was actually a bit surprised that the little man could reach his head that high. Larry had probably climbed onto his desk to do so, thought Josh. He had that faintly devious half-grin stuck on his face, as he always did when confronting his favourite nemesis.

Recognition probes: *Josh started the code/stated typing the code/started reading the code.*

(24)
Fred was thrilled that the summer had finally arrived and couldn't wait to start spending time outside. Fred even relished doing summer chores, which was fortunate because his front yard needed attention. The grass had grown so long that their home had lost all curb appeal. Hooked up to an extension cord from the garage, *Fred started the lawn*. | Over in the driveway, his 8 year old daughter Sam and her pet dog Pugly were supervising a pink lemonade stand. Fred was proud to see his daughter, a chip off the old block, with some entrepreneurial initiative. That was more than he could say for himself on this beautiful Tuesday morning. Fred was the proprietor of a chain of jewellery outlets in the broader metropolitan area. He had built up his business to the point that he could take such liberties and rely on his staff to handle the everyday grunt work. Recognition probes: *Fred started the lawn/started mowing the lawn/started uprooting the lawn.*
