# Supplementary material for: Context Breeds False Memories for Indeterminate Sentences
Source: Front Psychol. 2021 Mar 12;12:616065. doi: 10.3389/fpsyg.2021.616065 (PMC7994259; doi:10.3389/fpsyg.2021.616065)
Supplement: Supplementary file 3 [file Data_Sheet_3.pdf]

frontiers

Supplementary Material S3

Context Breeds False Memories for Indeterminate Sentences

Levi Riven[1] & Roberto G. de Almeida[1*]

*** Correspondence:**
Roberto G. de Almeida
roberto.dealmeida@concordia.ca

Means (SDs) for recognition accuracy and response confidence in Experiment 2. See text for discussion, and Figure 1.

	Proportion Correct	Response Confidence
Immediate Testing (0 s)		
Indeterminate (yes)	0.95 (.22)	6.94 (.42)
Biased foil (no)	0.94 (.23)	6.87 (.62)
Non-biased foil (no)	0.99 (.08)	6.94 (.45)
Delayed Testing (25 s)		
Indeterminate (yes)	0.49 (.50)	4.87 (1.62)
Biased foil (no)	0.51 (.50)	4.94 (1.63)
Non-biased foil (no)	0.90 (.31)	5.68 (1.78)
